# Associations of Appetitive Traits and Parental Feeding Style With Diet Quality During Early Childhood

**DOI:** 10.1016/j.jand.2024.02.004

**Published:** 2024-02-05

**Authors:** Jenna R. Cummings, Leah M. Lipsky, Myles S. Faith, Tonja R. Nansel

**Affiliations:** Department of Psychology; Social and Behavioral Sciences Branch, Division of Population Health Research, *Eunice Kennedy Shriver* National Institute of Child Health and Human Development; Department of Counseling, School, and Educational Psychology, Graduate School of Education, University at Buffalo–SUNY; Social and Behavioral Sciences Branch, Division of Population Health Research, *Eunice Kennedy Shriver* National Institute of Child Health and Human Development, Bethesda, MD

**Keywords:** Authoritarian, Appetitive traits, Diet quality, Early childhood, Parental feeding style

## Abstract

**Background:**

Appetitive traits and parent feeding styles are associated with body mass index in children, yet their associations with child diet quality are unclear.

**Objective:**

The objective was to examine relations of appetitive traits and parental feeding style with diet quality in 3.5-year-old children.

**Design:**

The study was a secondary, cross-sectional analysis of data from Sprouts, a follow-up study of the Pregnancy Eating Attributes Study (PEAS). Birthing parents completed the Child Eating Behavior Questionnaire, Caregiver’s Feeding Styles Questionnaire, and proxy 24-hour dietary recalls for their children from February 2019 to December 2020.

**Participants/setting:**

Participants were 162 birthing parents (early pregnancy BMI ≥ 18.5 and absence of preexisting diabetes, any medical condition contraindicating study participation, self-reported eating disorder, or medications that could affect diet or weight) and their children living in North Carolina.

**Main outcome measures:**

Healthy Eating Index—2015 (HEI-2015) total scores were calculated.

**Statistical analyses performed:**

Path modeling was conducted using PROC CALIS with full information maximum likelihood (FIML) to account for missing data (< 2% of all data in dataset). Associations of child appetitive traits and parental feeding style with child HEI-2015 scores, adjusting for exclusive breastfeeding duration and household income–poverty ratio, were examined. Tests of simple effects were conducted in subsamples split by parental feeding style. Hypotheses were formulated during data collection.

**Results:**

A 1-standard deviation (SD) greater food fussiness was associated with a 2.4-point lower HEI-2015 total score (*P* = .02; 95% confidence interval [CI] [−4.32, −0.48]) in children. When parental feeding style was authoritarian, a 1-SD greater food responsiveness was associated with a 4.1-point higher HEI-2015 total score (*P* = .007; 95% CI [1.12, 7.01]) in children. When parental feeding style was authoritative, a 1-SD greater slowness in eating was associated with a 5.8-point lower HEI-2015 total score (*P* =.01; 95% CI [−10.26, −1.33]) in children.

**Conclusions:**

Parental feeding style may modify the association of appetitive traits with diet quality in young children. Future research could determine whether matching parent feeding styles to child appetitive trait profiles improves child diet quality.

EVEN AT AGES 2 TO 5 YEARS, THE DIET QUALITY OF most American children is lacking, with inadequate intake of vegetables and whole grains and excess intake of refined grains, saturated fat, sodium, and added sugars.^1^ Lower diet quality during childhood is associated with elevated blood pressure, increased risk of metabolic syndrome, and worse quality of life.^2^ Given that parents are the main providers of food to children of young ages,^3^ examining parent and child behaviors associated with lower diet quality during early childhood may reveal intervention targets.

Child appetitive traits (ie, enduring predispositions toward food, also known as “child eating behaviors”) and parent feeding styles (ie, parents’ demandingness and responsiveness to their child’s eating behavior) have been associated with child body mass index (BMI)^4,5^; however, their relations with child diet quality are unclear. In young children, lower diet quality has consistently been associated with greater food fussiness, whereas associations with enjoyment of food, desire to drink, satiety responsiveness, and emotional undereating are mixed.^6-8^ Diet quality was not statistically significantly associated with food responsiveness, emotional overeating, or slowness in eating.^7,8^ Only one study has examined associations of parental feeding style with diet quality in young children, showing that an authoritative (ie, high demandingness and responsiveness) vs authoritarian (ie, high demandingness and low responsiveness) feeding style was associated with higher child diet quality at a single meal, with no differences compared with indulgent (ie, low demandingness and high responsiveness) and uninvolved (ie, low demandingness and responsiveness) feeding styles.^9^ Thus, there are knowledge gaps in understanding the relations of child appetitive traits and parent feeding styles with child diet quality.

Parent feeding styles may strengthen or weaken the behavioral expression of appetitive traits in children, thereby modifying the association of appetitive traits with diet quality. This idea is supported by research on general parenting style (ie, parents’ demandingness and responsiveness to their child’s behavior generally vs to their child’s eating behavior).^10^ Specifically, an uninvolved general parenting style strengthened obesity risk in children with greater food responsiveness, desire to drink, and emotional overeating, and an authoritative general parenting style weakened relations of food fussiness with lower fruit intake in children.^10^ To our knowledge, no study has examined whether parental feeding style—a more proximal indicator of parent–child eating interactions than general parenting style^5^—modifies relations of appetitive traits with diet quality in children.

The objective of the current study was to examine relations of child appetitive traits and parental feeding style with diet quality in 3.5-year-old children. First, based on research on associations of appetitive traits with adiposity,^4^ we hypothesized that food responsiveness, enjoyment of food, desire to drink, and emotional overeating, known as “food-approach” traits, would be associated with lower diet quality in children, whereas satiety responsiveness, slowness in eating, and emotional undereating, known as “food-avoidant” traits, would be associated with higher diet quality. Although food fussiness is considered a food-avoidant trait, we hypothesized food fussiness would be associated with lower diet quality consistent with prior findings.^6-8^ Second, based on findings from the prior study on associations of parental feeding style with diet quality in young children,^9^ we hypothesized that an authoritative feeding style would be associated with higher child diet quality compared with authoritarian, indulgent, and uninvolved feeding styles. Third, we hypothesized that an authoritative feeding style would weaken relations of greater child food-approach traits and food fussiness with lower child diet quality and strengthen relations of greater child food-avoidant traits with higher child diet quality relative to authoritarian, indulgent, and uninvolved feeding styles.

## MATERIALS AND METHODS

### Participants and Procedure

Data are from Sprouts, a follow-up study of the Pregnancy Eating Attributes Study (PEAS), which examined reward-related eating during pregnancy and postpartum in pregnant people in Chapel Hill, NC.^11^ Pregnant people (≤12 weeks’ gestation) were identified through the electronic medical records database and recruited from November 2014 to December 2016 through a university-based obstetrics hospital. Patients with early pregnancy BMI ≥ 18.5 were eligible if there was absence of preexisting diabetes, any medical condition contraindicating study participation, self-reported eating disorder, and medications that could affect diet or weight. Full inclusion and exclusion criteria and a flow diagram of the number of participants in PEAS at each stage were previously published,^12^ indicating 321 birthing parents completed the study through 1 year postpartum.

Sprouts began at child age 3.5 years and will continue with annual assessments of parent–child dyads through child age 7 years. Birthing parents who completed PEAS and provided consent for future contact were recruited for the first Sprouts assessment from February 2019 to December 2020; exclusion criteria were child neurocognitive disability or attention deficit/hyperactivity disorder. A total of 162 parent–child dyads enrolled in Sprouts via birthing parents providing informed consent for themselves and their children at child age 3.5 years.

Procedures were approved by the University of North Carolina Institutional Review Board (study #18-2030) and were in accordance with the ethical standards of the Helsinki Declaration of 1975 as revised in 1983. Initial study procedures for the child age 3.5 years assessment included in-person assessments, with 132 parent–child dyads completing in this manner; however, because of COVID-19 shutdowns, 30 parent–child dyads completed remotely. The current study was a secondary analysis using all available data. Hypotheses were formulated during data collection.

## MEASURES

### Dependent Variables

#### Child Diet Quality.

Dietary intake data were collected and analyzed using Nutrition Data System for Research software version 20,^13^ developed by the Nutrition Coordinating Center, University of Minnesota, Minneapolis, MN.^14^ Using the software, registered dietitians administered two proxy 24-hour dietary recalls, which are the most valid parent-report method for estimating intake in children ages 4 to 11.^15^ For parent–child dyads that completed an in-person assessment, one proxy 24-hour dietary recall was conducted at the assessment, and one was conducted remotely by phone approximately 10 days later. For parent–child dyads that completed remotely because of COVID-19 shutdowns, both 24-hour dietary recalls were conducted remotely by phone approximately 10 days apart.

Birthing parents reported to the interviewers all foods and beverages the child consumed from midnight to midnight the previous day, including preparation method, time of day consumed, food source, and portion size. To address reporting errors in dietary assessment, immediately after the recall, interviewers resolved any errors or unknown foods by asking the parent follow-up questions (eg, “Can you think of anything else your child ate or drank yesterday that we have not put on the list?”) or selecting defaults provided in Nutrition Data System for Research. Additionally, 25% of records as well as records with data 1 standard deviation (SD) above or below the mean daily energy for children (92 records) were reviewed for potential errors after completion of all recalls. Four records were modified by changing questionable food amounts to match interviewer notes or another recall from the participant.

The Healthy Eating Index—2015 (HEI-2015),^16^ a measure reflecting adherence to the 2015–2020 Dietary Guidelines for Americans,^17^ was calculated using the simple scoring algorithm method.^18^ Before applying the algorithm, data from multiple recalls were summed across all days per person. Three HEI-2015 scores were calculated: (1) the HEI-2015 adequacy score, which ranges from 0 to 60 and is calculated by summing 9 “adequacy” components (total fruits, whole fruits, total vegetables, greens and beans, whole grains, dairy, total protein foods, seafood and plant proteins, fatty acids); (2) the HEI-2015 moderation score, which ranges from 0 to 40 and is calculated by summing “moderation” components (refined grains, sodium, added sugars, saturated fats); and (3) the HEI-2015 total score, which ranges from 0 to 100 and is calculated by summing all 13 components. For all HEI-2015 scores, higher values indicate closer adherence to dietary guidelines.

### Independent Variables

#### Child Appetitive Traits.

Birthing parents completed the 35-item Child Eating Behavior Questionnaire,^19^ which measures 8 appetitive traits in children: food responsiveness (eg, “If given the chance, my child would always have food in his/her mouth”; 5 items; *α* = .61), enjoyment of food (eg, “My child enjoys eating”; four items; *α* = .65), desire to drink (eg, “My child is always asking for a drink”; 3 items, *α* = .89), emotional overeating (eg, “My child eats more when worried”; 4 items; *α* = .64), satiety responsiveness (eg, “My child gets full before his/her meal is finished”; 5 items; *α* = .69), slowness in eating (eg, “My child eats more and more slowly during the course of a meal”; 4 items; *α* = .68), emotional undereating (eg, “My child eats less when upset”; 4 items, *α* = .69), and food fussiness (eg, “My child refuses new foods at first”: 6 items; *α* = .91). Parents rated items on a 5-point Likert scale (1 = “Never,” 5 = “Always”). Items were reverse scored where appropriate, and items for each appetitive trait were averaged. Higher scores indicate greater food responsiveness, enjoyment of food, desire to drink, emotional overeating, satiety responsiveness, emotional undereating, and food fussiness and slower eating rates.

#### Parental Feeding Style.

Birthing parents completed the 19-item Caregiver’s Feeding Styles Questionnaire,^20^ which measures demandingness and responsiveness with regard to child feeding. Sample items include, “How often during the dinner meal do YOU tell the child to eat at least a little bit of food on his or her plate?” and “How often during the dinner meal do YOU allow the child to choose the foods he or she wants to eat for dinner from foods already prepared?” Parents rated items on a 5-point Likert scale (1 = “Never,” 5 = “Always”). All items assess demandingness, 7 items assess child-centered approaches (eg, asking questions, providing choice), and 12 items assess parent-centered approaches (eg, showing disapproval, providing rewards). Demandingness is calculated as the mean of all items, and responsiveness is calculated by scoring a ratio of the mean of the 7 child-centered approaches items to the mean of all items.^20^ Parental feeding style was based on median splits for demandingness and responsiveness: uninvolved (<2.70 demandingness, <1.09 responsiveness), authoritarian (≥2.70 demandingness, <1.09 responsiveness), indulgent (<2.70 demandingness, ≥1.09 responsiveness), and authoritative (≥2.70 demandingness, ≥1.09 responsiveness).

### Potential Confounders

Breastfeeding duration and household income were considered as confounders because of their hypothesized causal effects on child appetitive traits^21,22^ and diet quality.^23,24^ At each PEAS postpartum visit, birthing parents reported infant age when they introduced different feeding modes.^25^ A continuous measure of exclusive breastfeeding duration was calculated as the number of months birthing parents breastfed their infant or fed their infant breastmilk from a bottle, with no formula or complementary food feeding. Birthing parents reported household income at child aged 3.5 years, and the income–poverty ratio was calculated by dividing total annual household income by the US Census Bureau 2020 poverty thresholds, accounting for household size and composition.^26^

### Demographics

Birthing parents reported their race (White, Black, Asian, or Other or Multi-Race) and ethnicity (Hispanic or Latino or Not Hispanic or Latino) and their child’s sex (male or female) via a questionnaire.

### Statistical Analysis

Statistical analysis was conducted in SAS 9.4.^27^ Bivariate correlations (uncorrected for multiple comparisons) confirmed statistically significant (*P* < .05) associations of exclusive breastfeeding duration and household income–poverty ratio with child appetitive traits and diet quality (Supplemental Table 1); these variables were therefore included as covariates in subsequent models.

Path modeling was conducted using PROC CALIS with full information maximum likelihood (FIML) estimation to account for missing data (<2% of all data used were missing). Dependent variables (HEI-2015 total, adequacy, and moderation scores) were separately modeled. Independent variables were entered as follows: Step 1 included covariates, child appetitive traits, and parental feeding style, and Step 2 added multiplicative interaction terms between child appetitive traits and parental feeding style. Separate models were estimated for each child appetitive trait. For ease of interpretation, child appetitive traits were z-scored. For parental feeding style, a set of three dummy codes for authoritarian, indulgent, and authoritative feeding styles with uninvolved feeding style as the referent group was created. Statistical significance for the main effects and interactions was set at *P* < .05.

Where interactions were statistically significant, tests of simple effects were conducted in subsamples split by parental feeding style. Because these were conducted in subsamples for interpretation of statistically significant interactions, statistical significance for the tests of simple effects was set at *P* ≤ .10.

## RESULTS

Table 2 presents descriptive statistics for demographics and variables of interest. For child appetitive traits, enjoyment of food showed the highest mean value, and emotional overeating showed the lowest mean value. The most common parental feeding style was indulgent, followed by authoritarian, authoritative, and uninvolved. Average household income–poverty ratio indicates an annual household income of $68,852.40 for a family of three or $83,054 for a family of 4.

Table 3 presents estimates from path models examining associations of child appetitive traits and parental feeding style (step 1), and their interactions (step 2), with child HEI-2015 total scores (see Supplemental Table 4 for estimates from path models examining associations with child HEI-2015 adequacy and moderation scores). Holding household income–poverty ratio and exclusive breastfeeding duration constant, a 1-SD greater enjoyment of food was associated with a 2-point higher HEI-2015 total score in children, whereas a 1-SD greater food fussiness was associated with a 2.4-point lower HEI-2015 total score, independent of parental feeding style. Other associations of child appetitive traits with child HEI-2015 scores independent of parental feeding style were not statistically significant. There were no statistically significant associations of parental feeding style with child HEI-2015 scores independent of child appetitive traits.

**Figure 1.**
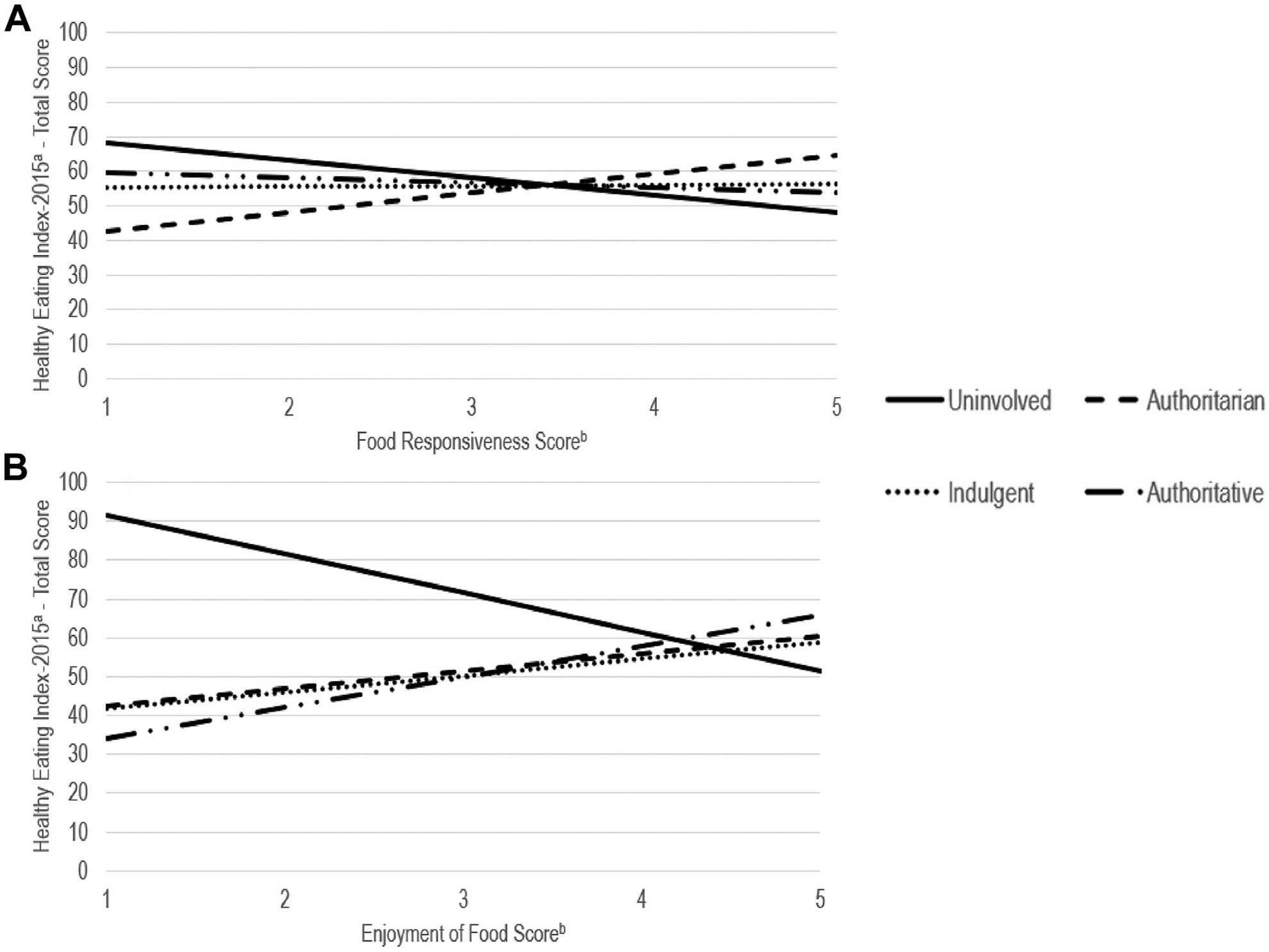
Simple slopes of child food-approach appetitive traits with child diet quality by parental feeding style in 162 parent–child dyads living in North Carolina; path modeling was conducted using PROC CALIS with full information maximum likelihood (FIML) to account for missing data (<2% of all data in dataset). The solid, round dot, dash, and long dash with round dots lines indicate simple slopes for uninvolved, indulgent, authoritarian, and authoritative feeding styles, respectively. (A) Healthy Eating Index—2015 total scores range from 0 to 100, with higher values indicating closer adherence to dietary guidelines. (B) Appetitive traits scores range from 1 to 5, with higher values indicating greater appetitive traits.

Parental feeding style statistically significantly modified associations of child food responsiveness and enjoyment of food with child HEI-2015 total scores. Figure 1 depicts simple slopes of these food-approach traits with child HEI-2015 total scores by parental feeding style, holding household income–poverty ratio and exclusive breastfeeding duration constant (see Supplemental Figure 2 for simple slopes with child HEI-2015 adequacy scores). A 1-SD greater food responsiveness was associated with a 3.7-point lower HEI-2015 total score (*P* = .10; 95% confidence interval [CI] [−8.15, .73]) in children when parental feeding style was uninvolved but was associated with a 4.1-point higher HEI-2015 total score (*P* = .007; 95% CI [1.12, 7.01)] when parental feeding style was authoritarian. Food responsiveness was not statistically significantly associated with HEI-2015 total scores in children when parental feeding style was indulgent or authoritative. A 1-SD greater enjoyment of food was associated with a 6.1-point lower HEI-2015 total score (*P* = .001; 95% CI [−9.61, −2.51]) in children when parental feeding style was uninvolved, but was associated with a 2.7-point higher HEI-2015 total score (*P* = .08; 95% CI [−0.30, 5.69]) when parental feeding style was authoritarian, and a 4.8-point higher HEI-2015 total score (*P* = .09; 95% CI [−0.72, 10.32]) when parental feeding style was authoritative. Enjoyment of food was not statistically significantly associated with HEI-2015 total scores in children when parental feeding style was indulgent.

**Figure 3.**
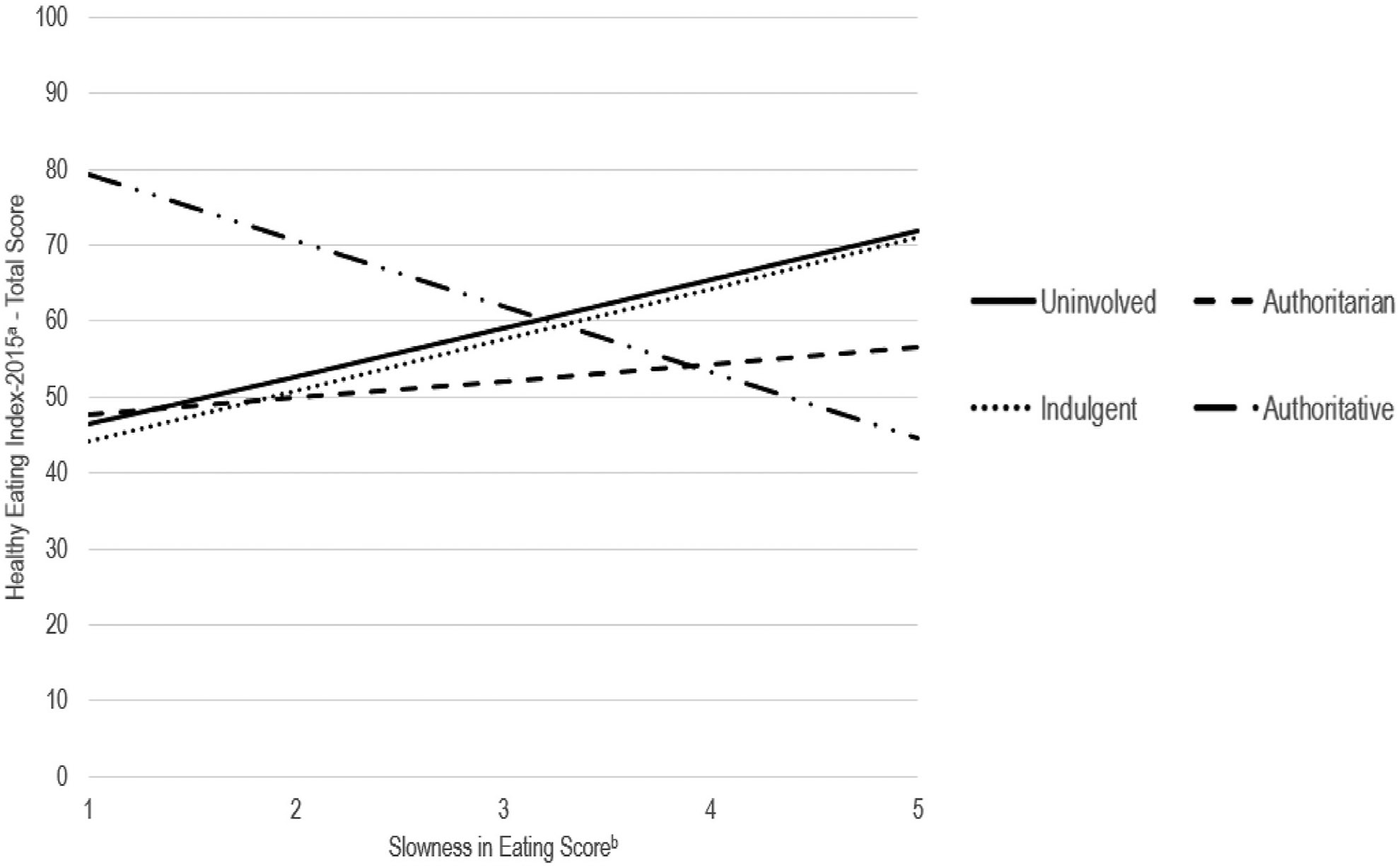
Simple slopes of child food-avoidant appetitive traits with child diet quality by parental feeding style in 162 parent–child dyads living in North Carolina; path modeling was conducted using PROC CALIS with full information maximum likelihood (FIML) to account for missing data (<2% of all data in dataset). The solid, round dot, dash, and long dash with round dots lines indicate simple slopes for uninvolved, indulgent, authoritarian, and authoritative feeding styles, respectively. (A) Healthy Eating Index-2015 total scores range from 0 to 100, with higher values indicating closer adherence to dietary guidelines. (B) Appetitive traits scores range from 1 to 5, with higher values indicating greater appetitive traits.

Parental feeding style statistically significantly modified associations of child slowness in eating with child HEI-2015 total scores. Figure 3 depicts simple slopes of this food-avoidant appetitive trait with child HEI-2015 total scores by parental feeding style, holding household income–poverty ratio and exclusive breastfeeding duration constant (Supplemental Fig 4 shows simple slopes with child HEI-2015 moderation scores). A 1-SD greater slowness in eating was associated with a 4.2-point higher HEI-2015 total score (*P* = .10; 95% CI [−0.76, 9.23]) in children when parental feeding style was uninvolved and a 4.5-point higher HEI-2015 total score (*P* = .02; 95% CI [0.65, 8.31]) when parental feeding style was indulgent. However, a 1-SD greater slowness in eating was associated with a 5.8-point lower HEI-2015 total score (*P* = .01; 95% CI [−10.26, −1.33]) in children when parental feeding style was authoritative. Slowness in eating was not statistically significantly associated with HEI-2015 total score in children when parental feeding style was authoritarian. Parental feeding style did not statistically significantly modify other associations of child appetitive traits with child HEI-2015 total scores.

## DISCUSSION

In a cross-sectional study of 162 young children from North Carolina, greater food fussiness was associated with lower diet quality, and parental feeding style modified associations of enjoyment of food, food responsiveness, and slowness in eating with diet quality. There were no statistically significant associations of desire to drink, emotional overeating and undereating, and satiety responsiveness with diet quality in children. Parental feeding style was not independently statistically significantly associated with child diet quality. These results suggest that some but not all appetitive traits are relevant to diet quality during early childhood, and that parental feeding style may alter the behavioral expression of child appetitive traits.

Regardless of parental feeding style, greater food fussiness was associated with lower diet quality, replicating prior findings in preschool children.^6-8^ Food fussiness has been associated with lower BMI in some studies (albeit, it has shown null associations with BMI in most prior studies).^4^ Given the robust association of greater food fussiness with lower diet quality in young children, separate effects of appetitive traits on diet quality and BMI should be considered when evaluating impact on overall child health. Food fussiness may compromise health through its adverse effects on diet quality, not BMI, during early childhood.

In contrast to the only previous study examining associations of parental feeding style with diet quality (observed at a single meal) in young children,^9^ in the current study, parental feeding style was not statistically significantly associated with child diet quality independent of child appetitive traits. However, parental feeding style modified associations of appetitive traits with diet quality in children. When birthing parents had an uninvolved feeding style (and to some extent an indulgent feeding style), directions of associations between appetitive traits and diet quality in children were consistent with those previously observed between appetitive traits and BMI^4^: greater food responsiveness and enjoyment of food were associated with lower diet quality, whereas greater slowness in eating was associated with higher diet quality. Uninvolved and indulgent feeding styles have been considered obesogenic,^5^ and when young children have a predisposition to eat more food, a lack of demands placed on a child’s eating may indeed be maladaptive. However, the current study findings suggest that when young children have a predisposition to eat less, the lack of demands on eating may be adaptive.

An authoritarian feeding style is also considered obesogenic.^5^ However, when birthing parents had an authoritarian feeding style, greater enjoyment of food and food responsiveness were associated with higher diet quality in children. These findings suggest that a parental feeding style characterized by higher demandingness, and using parent-centered approaches, may deter or reverse the detrimental association of food-approach traits with diet quality in young children. Although surprising, these findings align with previous research showing that an authoritarian parental feeding style at child ages 4 to 5 years predicted greater child satiety responsiveness at child ages 7 to 9 years,^28^ and an authoritarian parental feeding style was associated with intake of fewer unhealthy snacks in adolescents ages 12 to 16 years.^29^ An authoritarian feeding style has been considered obesogenic predominately because of positive associations between an authoritarian general parenting style and BMI in adolescents and young adults^5^ (an authoritarian feeding style was not associated with changes in BMI in a sample of young children^30^). Although the measure of parent feeding styles overlaps conceptually with general parenting style, the degree of concordance of these constructs is unknown. Possibly high demandingness and low responsiveness proximal to parent–child eating interactions may assist children for whom regulating intake of highly palatable foods is difficult.^29^ It is also possible that the potential benefits vs harms of an authoritarian parental feeding style change as children develop autonomy and need to rely on their satiety cues and self-regulation rather than external cues such as parent feeding.^31^ Longitudinal research from childhood to young adulthood could reveal whether relations of appetitive traits and parent feeding styles with diet quality in children change over time.

In children with birthing parents who had an authoritative feeding style, the feeding style considered optimal,^5^ greater enjoyment of food was associated with higher diet quality, but greater slowness in eating was associated with lower diet quality. Although these mixed findings were unexpected, they suggest that the strengths and weaknesses of an authoritative feeding style (like other parent feeding styles) are dependent on a child’s predispositions. Possibly a parental feeding style characterized by greater demands on the child to eat and by using more child-focused approaches to encourage eating—while beneficial when children demonstrate greater enjoyment of food—may impede children’s tendencies to self-regulate their dietary intake through slowness in eating. The prior study showing that an authoritative vs authoritarian feeding style was associated with higher child diet quality did not account for child appetitive traits,^9^ whereas the current study examined the independent and interactive associations of child appetitive traits and parental feeding style with child diet quality. Future research should continue examining synergistic associations with child health outcomes because this approach more accurately reflects family dynamics.

Child desire to drink, emotional overeating and undereating, and satiety responsiveness have been associated with BMI in children.^4,5^ However, associations of child desire to drink, emotional overeating and undereating, and satiety responsiveness with child diet quality have been mixed in prior studies.^7,8^ Divergence between associations with child BMI and diet quality in the literature may reflect contributions of diet quality vs total energy intake to BMI or nondietary variables affecting BMI.

There are strengths and limitations of the current study to consider when interpreting findings. A systematic review indicated proxy 24-hour dietary recall (vs food frequency questionnaires and diet history) estimates total energy intake of children most similarly to total energy expenditure measured by doubly labeled water^15^; internal validity was therefore strengthened by assessing child dietary intake with this method. The measurement and adjustment for potential confounds including household income and breastfeeding duration (measured during postpartum) further strengthened internal validity. Sociodemographic characteristics of the sample are consistent with those from Chapel Hill, North Carolina, which limits generalizability at the national and global level. Although mean child appetitive traits and parental feeding styles were comparable to those of prior studies,^7,20,32,33^ the families in the current study had higher household incomes than the families in most prior studies on parental feeding styles.^9,28,30,33^ Although the current study analyses adjusted for household income, possibly household income plays a role in associations among appetitive traits, parental feeding style, and diet quality in children. Future research could directly test for differences related to parent feeding styles between families with lower vs higher incomes. In the current study, feeding style was only assessed in birthing parents, and the feeding style of a co-parent may differ; future research could assess both parents’ feeding styles. Given the cross-sectional design, causal conclusions cannot be made.

## CONCLUSIONS

Parental feeding style may modify the association of appetitive traits with diet quality in young children. Authoritative and authoritarian feeding styles may reduce the negative effects of greater food-approach traits, specifically food responsiveness and enjoyment of food, and an authoritative feeding style may reduce the positive effects of slowness in eating, a food-avoidant trait. Future research could determine whether matching parent feeding styles to appetitive trait profiles in children increases child diet quality. Further investigating interactive associations with diet quality in children may reveal how parent–child dynamics can be leveraged to promote child health.

## Supplementary Material

1

## Figures and Tables

**Table 2.** Descriptive statistics for demographics, child diet quality, child appetitive traits, and parental feeding style in 162 parent–child dyads living in North Carolina

	n	Mean (SD) or %
**Demographics** ^a^		
Birthing parent race		
White	123	76.4%
Black	19	11.8%
Asian	7	4.3%
Other or multi-race	12	7.5%
Birthing parent ethnicity		
Hispanic or Latino	16	9.9%
Not Hispanic or Latino	145	90.1%
Child sex		
Female	83	51.6%
Male	78	48.4%
Exclusive breastfeeding duration (months)	154	2.09 (2.63)
Household income–poverty ratio	157	3.17 (1.22)
< 1	9	5.7%
1–1.99	22	14.0%
2–2.99	28	17.8%
3–3.99	28	17.8%
4–4.99	57	36.3%
≥5	13	8.3%
**Child diet quality** ^a^		
HEI-2015^b^ Total score (0–100)	161	59.78 (12.14)
HEI-2015^b^ Adequacy score (0–60)	161	34.51 (8.17)
HEI-2015^b^ Moderation score (0–40)	161	25.27 (5.53)
**Child appetitive traits** ^a^		
Food responsiveness (1–5)	160	2.48 (0.73)
Enjoyment of food (1–5)	159	3.80 (0.61)
Desire to drink (1–5)	160	2.99 (1.02)
Emotional over-eating (1–5)	160	1.61 (0.53)
Satiety responsiveness (1–5)	160	3.07 (0.53)
Slowness in eating (1–5)	160	3.08 (0.67)
Emotional undereating (1–5)	160	3.01 (0.89)
Food fussiness (1–5)	160	2.87 (0.77)
**Parental feeding style** ^a^		
Authoritative	26	16.3%
Authoritarian	54	33.8%
Indulgent	59	36.9%
Uninvolved	21	13.1%

a Some data are missing for each variable (<2% of all data in dataset).

b HEI-2015 = Healthy Eating Index—2015.

**Table 3.** Relations of child appetitive traits, parental feeding style, and their interaction with child diet quality in 162 parent–child dyads living in North Carolina

		HEI-2015^a,b^Total Score		
		*b ± SE*	*P*	95% CI (lower, upper)
Model 1^b,c,d^	Food responsiveness	0.67 ± 0.95	.482	(−1.19, 2.52)
	PFS authoritative	−1.90 ± 3.48	.586	(−8.71, 4.92)
	PFS authoritarian	−0.42 ± 3.06	.892	(−6.41, 5.58)
	PFS indulgent	0.37 ± 3.00	.902	(−5.51, 6.25)
	Food responsiveness × PFS authoritative	3.29 ± 3.68	.370	(−3.91, 10.50)
	Food responsiveness × PFS authoritarian	8.05 ± 3.32	.015	(1.55, 14.55)
	Food responsiveness × PFS indulgent	4.37 ± 3.21	.173	(−1.92, 10.66)
Model 2^b,c,d^	Enjoyment of food	1.96 ± 0.99	.048	(0.01, 3.91)
	PFS authoritative	−2.19 ± 3.44	.525	(−8.93, 4.55)
	PFS authoritarian	0.19 ± 3.04	.950	(−5.76, 6.15)
	PFS indulgent	−0.51 ± 2.99	.865	(−6.37, 5.35)
	Enjoyment of food × PFS authoritative	7.81 ± 3.74	.037	(0.49, 15.14)
	Enjoyment of food × PFS authoritarian	5.95 ± 3.02	.049	(0.02, 11.87)
	Enjoyment of food × PFS indulgent	5.67 ± 2.99	.058	(−0.18, 11.53)
Model 3^b,c,d^	Desire to drink	−1.01 ± 1.07	.344	(−3.10, 1.08)
	PFS authoritative	−1.38 ± 3.49	.693	(−8.22, 5.47)
	PFS authoritarian	−0.08 ± 3.07	.980	(−6.10, 5.94)
	PFS indulgent	0.18 ± 3.00	.953	(−5.70, 6.05)
	Desire to drink × PFS authoritative	−0.91 ± 3.26	.781	(−7.30, 5.48)
	Desire to drink × PFS authoritarian	0.58 ± 3.07	.849	(−5.43, 6.59)
	Desire to drink ± PFS indulgent	1.27 ± 3.11	.683	(−4.81, 7.36)
Model 4^b,c,d^	−motional over-eating	−0.15 ± 0.96	.872	(−2.04, 1.73)
	PFS authoritative	−1.73 ± 3.50	.620	(−8.60, 5.13)
	PFS authoritarian	−0.32 ± 3.11	.919	(−6.41, 5.78)
	PFS indulgent	0.29 ± 3.01	.922	(−5.60, 6.19)
	Emotional overeating × PFS authoritative	3.10 ± 3.58	.389	(−3.92, 10.11)
	Emotional overeating × PFS authoritarian	3.14 ± 3.47	.366	(−3.67, 9.95)
	Emotional overeating ± PFS indulgent	1.94 ± 3.24	.548	(−4.40, 8.29)
Model 5^b,c,d^	Satiety responsiveness	0.31 ± 1.00	.752	(−1.64, 2.26)
	PFS authoritative	−1.89±3.49	.587	(−8.73, 4.94)
	PFS authoritarian	−0.64 ± 3.14	.840	(−6.79, 5.52)
	PFS indulgent	0.23 ± 3.00	.938	(−5.65, 6.12)
	Satiety responsiveness × PFS authoritative	−3.93 ± 3.56	.270	(−10.91, 3.05)
	Satiety responsiveness × PFS authoritarian	−0.49 ± 2.82	.868	(−6.02, 5.04)
	Satiety responsiveness × PFS indulgent	1.20 ± 3.01	.690	(−4.71, 7.11)
Model 6^b,c,d^	Slowness in eating	1.15 ± 0.98	.243	(−0.78, 3.08)
	PFS authoritative	−1.56 ± 3.47	.653	(−8.35, 5.24)
	PFS authoritarian	−0.57 ± 3.05	.853	(−6.55, 5.41)
	PFS indulgent	0.72 ± 3.01	.810	(−5.17, 6.62)
	Slowness in eating × PFS authoritative	−7.81 ± 3.84	.042	(−15.33, −0.29)
	Slowness in eating × PFS authoritarian	−0.60 ± 3.44	.861	(−7.34, 6.14)
	Slowness in eating × PFS indulgent	2.06 ± 3.52	.559	(−4.84, 8.95)
Model 7^b,c,d^	Emotional undereating	−0.25 ± 1.01	.803	(−2.22, 1.72)
	PFS authoritative	−1.69 ± 3.50	.629	(−8.55, 5.17)
	PFS authoritarian	−0.28 ± 3.11	.927	(−6.38, 5.81)
	PFS indulgent	0.20 ± 3.01	.946	(−5.69, 6.10)
	Emotional undereating × PFS authoritative	−6.29 ± 4.60	.171	(−15.31, 2.72)
	Emotional undereating × PFS authoritarian	−1.81 ± 3.10	.560	(−7.89, 4.28)
	Emotional undereating × PFS indulgent	0.14 ± 2.97	.963	(5.69, 5.97)
Model 8^b,c,d^	Food fussiness	−2.40 ± 0.98	.015	(−4.32, −0.48)
	PFS authoritative	−1.71 ± 3.41	.616	(−8.40, 4.98)
	PFS authoritarian	0.90 ± 3.05	.768	(−5.08, 6.88)
	PFS indulgent	−0.29 ± 2.96	.922	(−6.08, 5.50)
	Food fussiness × PFS authoritative	−3.39 ± 4.10	.408	(−11.42, 4.64)
	Food fussiness × PFS authoritarian	2.54 ± 3.34	.446	(−3.99, 9.08)
	Food fussiness × PFS indulgent	3.87 ± 3.23	.230	(−2.45, 10.20)

a HEI-2015 = Healthy Eating Index-2015.

b Path modeling was conducted using PROC CALIS with full information maximum likelihood (FIML) to account for missing data (<2% of all data in dataset).

c For parental feeding style (PFS), a set of three dummy codes for authoritarian, indulgent, and authoritative feeding styles with uninvolved feeding style as the referent group and multiplicative interaction terms between child appetitive traits and parental feeding style were created. Path modeling adjusted for household income–poverty ratio and exclusive breastfeeding duration. Estimates for main effects of child appetitive traits and parental feeding style are from step 1 (without multiplicative interaction terms). Estimates for interactions between child appetitive traits and parental feeding style are from step 2.

d Statistical significance for the main effects and interactions was set at *P* < .05. Where interactions were statistically significant, tests of simple effects were conducted in subsamples split by parental feeding style. Because these were conducted in subsamples for interpretation of statistically significant interactions, statistical significance for the tests of simple effects was set at *P* ≤ .10.

## RESEARCH SNAPSHOT

### Research Question:

How does parental feeding style modify the association of appetitive traits with diet quality in young children?

### Key Findings:

In this analysis of cross-sectional data from 162 parent–child dyads living in North Carolina, greater food responsiveness was significantly associated with higher diet quality in children when parental feeding style was authoritarian. Greater slowness in eating was significantly associated with lower diet quality in children when parental feeding style was authoritative. Regardless of parental feeding style, children with greater food fussiness had significantly lower diet quality.
