# Supplementary material for: Associations of Appetitive Traits and Parental Feeding Style With Diet Quality During Early Childhood
Source: J Acad Nutr Diet. Author manuscript; Available in PMC 2025 Jul 1. (PMC12045477; doi:10.1016/j.jand.2024.02.004)
Supplement: 1 [file NIHMS2071650-supplement-1.pdf]

Supplemental Table 1. Bivariate correlations testing for potential confounders in associations between appetitive traits and diet quality in 162 children living in North Carolina

	1.	2.	3.	4.	5.	6.	7.	8.	9.	10.	11.	12.
**Potential Confounders**[a]												
1. Exclusive breastfeeding duration (months)	—	—	—	—	—	—	—	—	—	—	—	—
2. Income−poverty ratio	.07	—	—	—	—	—	—	—	—	—	—	—
**Child appetitive traits**[a]												
3. Food responsiveness (1−5)	.01	−.11	—	—	—	—	—	—	—	—	—	—
4. Enjoyment of food (1−5)	.11	−.08	.51***	—	—	—	—	—	—	—	—	—
5. Desire to drink (1−5)	−.20*	−.37***	.23***	−.01	—	—	—	—	—	—	—	—
6. Emotional overeating (1−5)	−.08	−.01	.55***	.14	.20*	—	—	—	—	—	—	—
7. Satiety responsiveness (1−5)	−.01	.18*	−.32***	−.47***	.02	−.13	—	—	—	—	—	—
8. Slowness in eating (1−5)	−.11	.09	−.03	−.27***	.17*	.06	.45***	—	—	—	—	—
9. Emotional undereating (1−5)	−.13	−.05	.13	−.10	.22**	.30***	.34***	.26***	—	—	—	—
10. Food fussiness (1−5)	−.08	.01	−.14	−.50***	.15	.07	.26***	.14	.17*	—	—	—
**Child Diet Quality**[a]												
11. HEI-2015[b] Total Score (0−100)	.21*	.09	.04	.16*	−.15	−.04	.03	.06	−.07	−.20*	—	—
12. HEI-2015[b] Adequacy Score (0−60)	.16	.16*	.05	.18*	−.18*	−.03	−.01	.02	−.11	−.23**	.93***	—
13. HEI-2015[b] Moderation Score (0−40)	.22**	−.04	.01	.08	−.07	−.05	.08	.09	.02	−.09	.83***	.55***

*P < .05; **P < .01; ***P < .001.

[a]Some data are missing for each variable (<2% of all data in dataset).

[b]HEI-2015 = Healthy Eating Index—2015.

Supplemental Table 4. Relations of child appetitive traits, parental feeding style, and their interaction with Child Healthy Eating Index—2015 adequacy and moderation scores in 162 parent–child dyads living in North Carolina

		HEI-2015[a] Adequacy Score			HEI-2015[a] Moderation Score		
		b ± *SE*	*P*	95% CI (lower, upper)	*b* ± *SE*	*P*	95% CI (lower, upper)
Model 1[b,c,d]	Food responsiveness	0.62 ± 0.64	.335	(−0.64, 1.87)	0.43 ± 0.12	.908	(−0.79, 0.89)
	PFS authoritative	−1.39 ± 2.35	.553	(−6.00, 3.21)	−0.53 ± 1.58	.735	(−3.62, 2.56)
	PFS authoritarian	−0.09 ± 2.07	.965	(−4.14, 3.96)	−0.33 ± 1.39	.811	(−3.05, 2.39)
	PFS indulgent	0.78 ± 2.03	.700	(−3.19, 4.76)	−0.39 ± 1.36	.774	(−3.06, 2.28)
	Food responsiveness × PFS authoritative	4.19 ± 2.46	.089	(−0.64, 9.02)	−0.83 ± 1.69	.622	(−4.14, 2.47)
	Food responsiveness × PFS authoritarian	6.52 ± 2.22	.003	(2.17, 10.88)	1.61 ± 1.52	.290	(1.37, 4.59)
	Food responsiveness × PFS indulgent	3.82 ± 2.15	.076	(−0.40, 8.04)	0.61 ± 1.47	.681	(−2.28, 3.49)
Model 2[b,c,d]	Enjoyment of food	1.62 ± 0.67	.015	(0.32, 2.93)	0.35 ± 0.46	.443	(−0.54, 1.24)
	PFS authoritative	−1.62 ± 2.31	.483	(−6.15, 2.91)	−0.59 ± 1.57	.706	(−3.68, 2.49)
	PFS authoritarian	0.42 ± 2.04	.839	(−3.59, 4.42)	−0.22 ± 1.39	.873	(−2.95, 2.50)
	PFS indulgent	0.05 ± 2.01	.981	(−3.89, 3.99)	-0.54 ± 1.37	.694	(−3.22, 2.14)
	Enjoyment of food × PFS authoritative	6.96 ± 2.47	.005	(2.13, 11.79)	0.92 ± 1.74	.596	(−2.48, 4.32)
	Enjoyment of food × PFS authoritarian	5.79 ± 1.99	.004	(1.89, 9.69)	0.21 ± 1.40	.882	(−2.54, 2.96)
	Enjoyment of food × PFS indulgent	5.03 ± 1.97	.011	(1.17, 8.90)	0.68 ± 1.39	.623	(−2.04, 3.40)
Model 3 [b,c,d]	Desire to drink	−0.76 ± 0.72	.295	(−2.17, 0.66)	−0.27 ± 0.49	.577	(−1.22, 0.68)
	PFS authoritative	−1.00 ± 2.36	.672	(−5.63, 3.63)	−0.40 ± 1.58	.800	(−3.50, 2.70)
	PFS authoritarian	0.14 ± 2.08	.944	(−3.93, 4.22)	−0.22 ± 1.39	.872	(−2.95, 2.50)
	PFS indulgent	0.60 ± 2.03	.766	(−3.37, 4.58)	−0.41 ± 1.36	.763	(−3.08, 2.25)
	Desire to drink × PFS authoritative	−0.51 ± 2.21	.819	(−4.84, 3.83)	−0.41 ± 1.48	.782	(−3.30, 2.49)
	Desire to drink × PFS authoritarian	0.04 ± 2.08	.986	(−4.03, 4.11)	0.54 ± 1.39	.700	(−2.19, 3.26)
	Desire to drink × PFS indulgent	0.69 ± 2.10	.743	(−3.43, 4.81)	0.59 ± 1.41	.675	(−2.17, 3.35)
Model 4 [b,c,d]	Emotional overeating	0.00 ± 0.65	.998	(−1.28, 1.27)	−0.16 ± 0.43	.721	(−1.01, 0.70)
	PFS authoritative	−1.30 ± 2.37	.582	(−5.95, 3.34)	−0.46 ± 1.58	.773	(−3.56, 2.65)
	PFS authoritarian	−0.08 ± 2.10	.971	(−4.20, 4.05)	−0.24 ± 1.41	.864	(−3.00, 2.52)
	PFS indulgent	0.68 ± 2.04	.737	(−3.30, 4.67)	−0.37 ± 1.36	.786	(−3.04, 2.30)
	Emotional overeating × PFS Authoritative	2.35 ± 2.41	.329	(−2.37, 7.07)	0.78 ± 1.61	.631	(−2.39, 3.94)
	Emotional overeating × PFS Authoritarian	1.75 ± 2.34	.454	(−2.83, 6.33)	1.39 ± 1.57	.374	(−1.68, 4.46)
	Emotional overeating × PFS indulgent	−0.15 ± 2.18	.944	(-4.42, 4.12)	2.07 ± 1.46	.156	(−0.79, 4.93)
Model 5[b,c,d]	Satiety responsiveness	−0.30 ± 0.67	.659	(−1.62, 1.02)	0.60 ± 0.45	.183	(−0.28, 1.48)
	PFS authoritative	−1.23 ± 2.36	.602	(−5.86, 3.39)	−0.69 ± 1.57	.661	(−3.77, 2.39)
	PFS authoritarian	0.12 ± 2.12	.953	(−4.04, 4.29)	−0.75 ± 1.41	.595	(−3.52, 2.02)
	PFS indulgent	0.71 ± 2.03	.728	(−3.28, 4.69)	−0.45 ± 1.35	.739	(−3.10, 2.20)

(*continued on next page*)

Supplemental Table 4. Relations of child appetitive traits, parental feeding style, and their interaction with Child Healthy Eating Index—2015 adequacy and moderation scores in 162 parent—child dyads living in North Carolina (*continued*)

		HEI-2015[a] Adequacy Score			HEI-2015[a] Moderation Score		
		b ± *SE*	*P*	95% CI (lower, upper)	*b* ± *SE*	*P*	95% CI (lower, upper)
	Satiety responsiveness × PFS authoritative	−3.21 ± 2.41	.183	(−7.92, 1.51)	−0.77 ± 1.61	.633	(−3.93, 2.39)
	Satiety responsiveness × PFS authoritarian	−0.19 ± 1.91	.920	(.−3.92, 3.54)	−0.31 ± 1.28	.810	(−2.81, 2.19)
	Satiety responsiveness × PFS indulgent	0.41 ± 2.04	.842	(−3.59, 4.40)	0.79 ± 1.36	.562	(−1.88, 3.46)
Model 6 [b,c,d]	Slowness in eating	0.37 ± 0.67	.580	(−0.94, 1.68)	0.77 ± 0.44	.083	(−0.10, 1.64)
	PFS authoritative	−1.22 ± 2.35	.605	(−5.83, 3.40)	−0.37 ± 1.56	.811	(−3.43, 2.69)
	PFS authoritarian	−0.12 ± 2.07	.952	(−4.19, 3.94)	−0.45 ± 1.37	.744	(−3.14, 2.25)
	PFS indulgent	0.84 ± 2.04	.683	(−3.17, 4.84)	−0.10 ± 1.36	.944	(−2.76, 2.56)
	Slowness in eating × PFS authoritative	−4.37 ± 2.62	.096	(−9.51, 0.78)	−3.48 ± 1.74	.045	(−6.88, -0.07)
	Slowness in eating × PFS authoritarian	−0.70 ± 2.35	.766	(−5.31, 3.91)	0.09 ± 1.56	.954	(−2.96, 3.14)
	Slowness in eating × PFS indulgent	1.60 ± 2.41	.506	(−3.11, 6.32)	0.50 ± 1.59	.753	(−2.62, 3.63)
Model 7 [b,c,d]	Emotional undereating	−0.60 ± 0.68	.378	(−1.93, 0.73)	0.33 ± 0.46	.467	(−0.56, 1.22)
	PFS authoritative	−1.05 ± 2.37	.656	(−5.69, 3.58)	−0.66 ± 1.58	.677	(−3.76, 2.44)
	PFS authoritarian	0.24 ± 2.10	.908	(−3.88, 4.36)	−0.52 ± 1.41	.710	(−3.28, 2.23)
	PFS indulgent	0.56 ± 2.03	.784	(−3.42, 4.54)	−0.34 ± 1.36	.803	(−3.00, 2.33)
	Emotional undereating × PFS authoritative	−5.02 ± 3.10	.106	(−11.09, 1.06)	−1.38 ± 2.09	.510	(−5.47, 2.71)
	Emotional undereating × PFS authoritarian	−1.33 ± 2.10	.526	(−5.44, 2.78)	−0.51 ± 1.41	.719	(−3.27, 2.25)
	Emotional undereating × PFS indulgent	−0.59 ± 2.01	.770	(−4.52, 3.34)	0.69 ± 1.35	.609	(−1.96, 3.34)
Model 8 [b,c,d]	Food fussiness	−2.00 ± 0.66	.002	(−3.28, −0.71)	−0.43 ± 0.45	.337	(−1.32, 0.45)
	PFS authoritative	−1.23 ± 2.29	.590	(−5.71, 3.25)	−0.50 ± 1.57	.748	(−3.58, 2.57)
	PFS authoritarian	1.01 ± 2.04	.621	(−3.00, 5.02)	−0.09 ± 1.40	.949	(−2.84, 2.66)
	PFS indulgent	0.22 ± 1.98	.911	(−3.66, 4.10)	−0.50 ± 1.36	.714	(−3.16, 2.17)
	Food fussiness × PFS authoritative	−1.74 ± 2.74	.523	(−7.12, 3.64)	−1.66 ± 1.90	.381	(−5.38, 2.06)
	Food fussiness × PFS authoritarian	2.42 ± 2.24	.278	(−1.96, 6.81)	0.15 ± 1.54	.924	(−2.88, 3.17)
	Food fussiness × PFS indulgent	2.77 ± 2.16	.200	(−1.47, 7.01)	1.13 ± 1.49	.450	(−1.80, 4.06)

[a]HEI-2015 = Healthy Eating Index-2015.
[b]Path modeling was conducted using PROC CALIS with full information maximum likelihood (FIML) to account for missing data (<2% of all data in dataset).
[c]For parental feeding style (PFS), a set of three dummy codes for authoritarian, indulgent, and authoritative feeding styles with uninvolved feeding style as the referent group and multiplicative interaction terms between child appetitive traits and parental feeding style were created. Path modeling adjusted for household income—poverty ratio and exclusive breastfeeding duration. Estimates for main effects of child appetitive traits and parental feeding style are from step 1 (without multiplicative interaction terms). Estimates for interactions between child appetitive traits and parental feeding style are from step 2.
[d]Statistical significance for the main effects and interactions was set at $P < .05$. Where interactions were statistically significant, tests of simple effects were conducted in subsamples split by parental feeding style. Because these were conducted in subsamples for interpretation of statistically significant interactions, statistical significance for the tests of simple effects was set at $P \leq .10$.

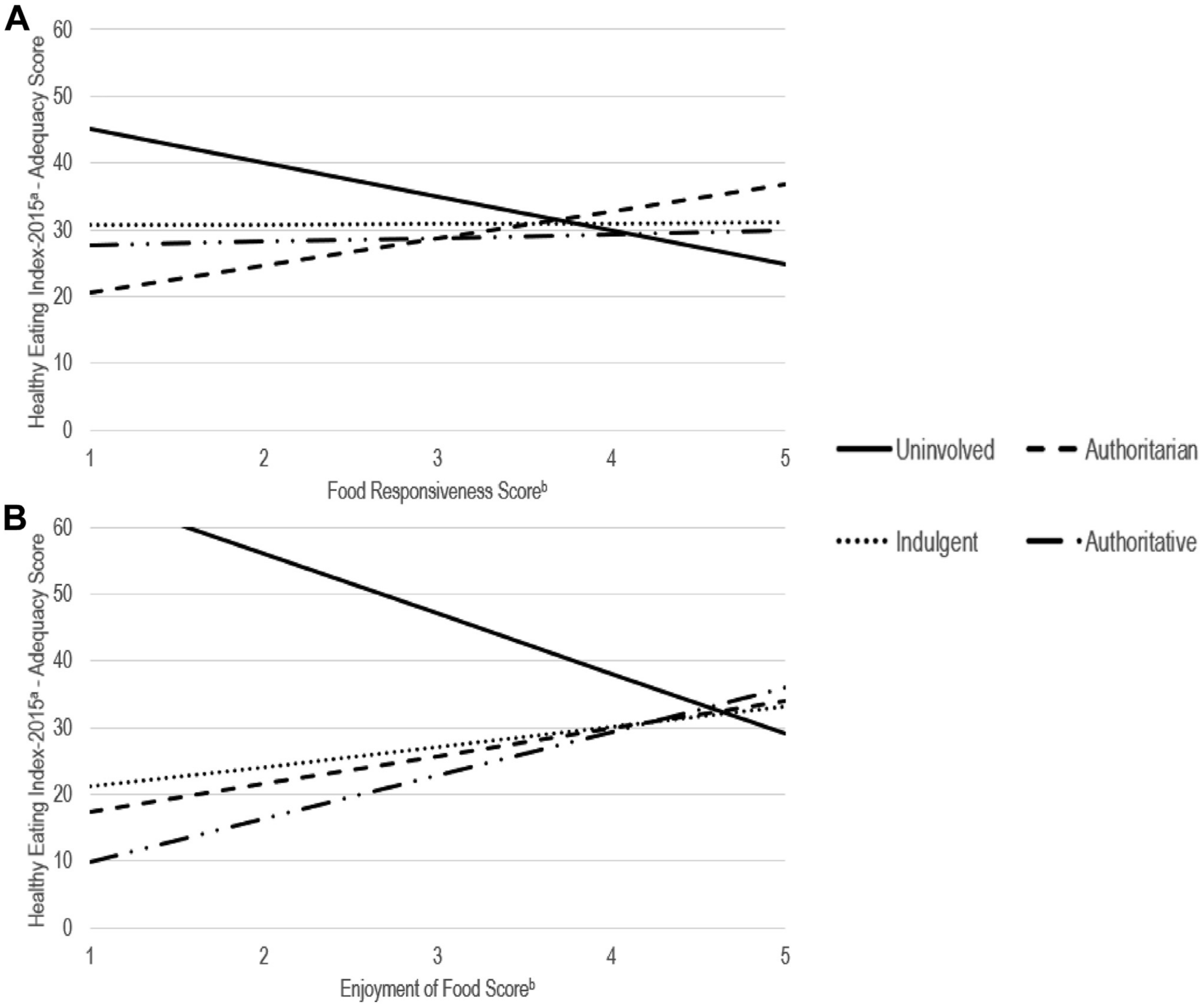

Supplemental Figure 2. Simple slopes of child food-approach appetitive traits with child Healthy Eating Index—2015 (HEI-2015) adequacy scores by parental feeding style in 162 parent−child dyads living in North Carolina; path modeling was conducted using PROC CALIS with full information maximum likelihood (FIML) to account for missing data (<2% of all data in dataset). The solid, round dot, dash, and long dash with round dots lines indicate simple slopes for uninvolved, indulgent, authoritarian, and authoritative feeding styles, respectively. Parental feeding style statistically significantly modified associations of child food responsiveness and enjoyment of food with child HEI-2015 adequacy (but not moderation) scores. Holding household income−poverty ratio and exclusive breastfeeding duration constant, a 1-SD greater food responsiveness was associated with a 3.7-point lower HEI-2015 adequacy score ($P = .06$; 95% CI [−7.52, 0.08]) in children when parental feeding style was uninvolved, but was associated with a 3-point higher HEI-2015 adequacy score ($P = .001$; 95% CI [1.16, 4.78]) when parental feeding style was authoritarian. Food responsiveness was not statistically significantly associated with HEI-2015 adequacy scores in children when parental feeding style was indulgent or authoritative. Holding household income−poverty ratio and exclusive breastfeeding duration constant, a 1-SD greater enjoyment of food was associated with a 5.5-point lower HEI-2015 adequacy score ($P = .001$; 95% CI [−8.53, −2.38]) in children when parental feeding style was uninvolved, but was associated with a 2.5-point higher adequacy score ($P = .006$; 95% CI [0.73, 4.31]) when parental feeding style was authoritarian, and a 4-point higher HEI-2015 adequacy score ($P = .02$; 95% CI [0.65, 7.25]) when parental feeding style was authoritative. Enjoyment of food was not statistically significantly associated with HEI-2015 adequacy scores in children when parental feeding style was indulgent. (A) Healthy Eating Index-2015 adequacy scores range from 0 to 60, with higher values indicating closer adherence to dietary guidelines. (B) Appetitive traits scores range from 1 to 5, with higher values indicating greater appetitive traits.

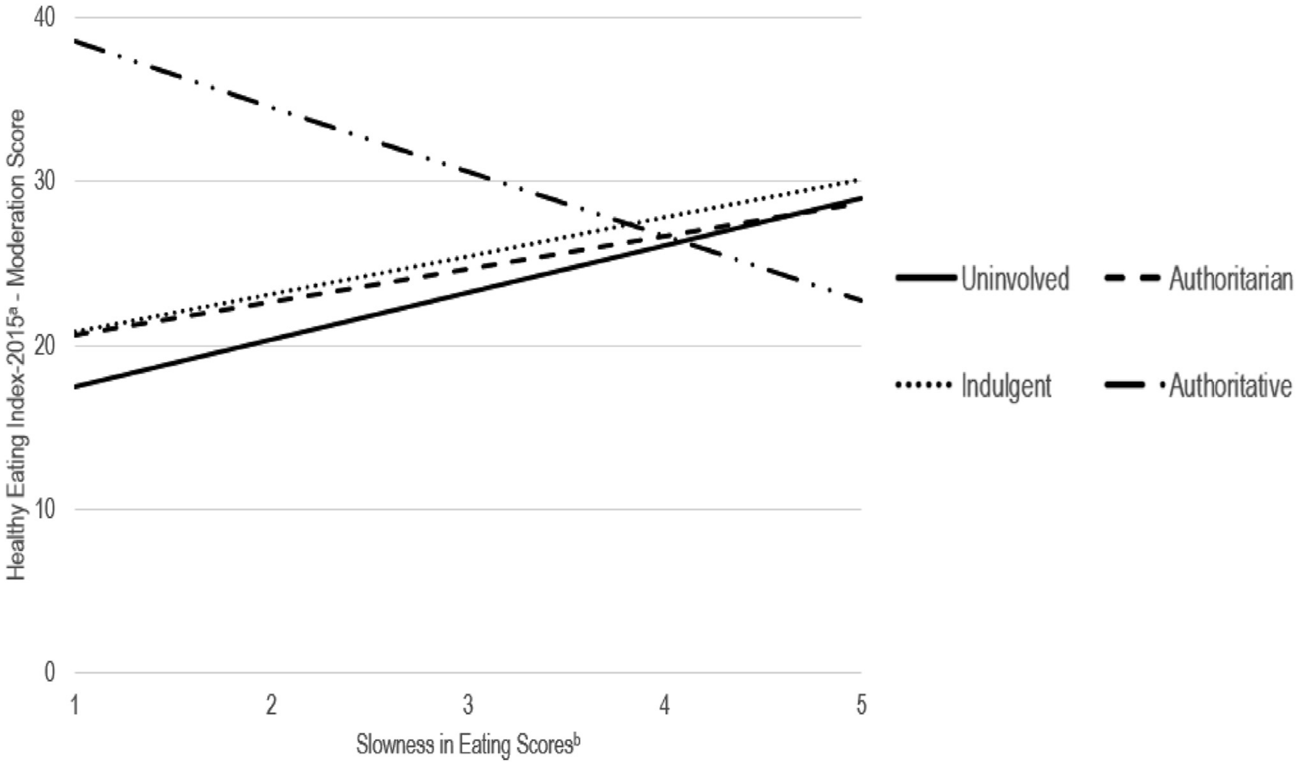

Supplemental Figure 4. Simple slopes of child food-avoidant appetitive traits with child Healthy Eating Index-2015 (HEI-2015) moderation scores by parental feeding style in 162 parent—child dyads living in North Carolina; path modeling was conducted using PROC CALIS with full information maximum likelihood (FIML) to account for missing data (< 2% of all data in dataset). The solid, round dot, dash, and long dash with round dots lines indicate simple slopes for uninvolved, indulgent, authoritarian, and authoritative feeding styles, respectively. Parental feeding style statistically significantly modified associations of child slowness in eating with child HEI-2015 moderation (but not adequacy) scores. Holding household income—poverty ratio and exclusive breastfeeding duration constant, a 1-SD greater slowness in eating was associated with a 1.9-point higher HEI-2015 moderation score (P = .09; 95% CI [−0.31, 4.15]) in children when parental feeding style was uninvolved, a 1.3-point higher HEI-2015 moderation score (P = .04; 95% CI [0.06, 2.61]) when parental feeding style was authoritarian, and a 1.6-point higher HEI-2015 moderation score (P = .08; 95% CI [−0.21, 3.33]) when parental feeding style was indulgent. However, a 1-SD greater slowness in eating was associated with a 2.6-point lower HEI-2015 moderation score (P = .01; 95% CI [−4.64, −0.61]) in children when parental feeding style was authoritative. (A) Healthy Eating Index-2015 moderation scores range from 0 to 40, with higher values indicating closer adherence to dietary guidelines. (B) Appetitive traits scores range from 1 to 5, with higher values indicating greater appetitive traits.
